# A comprehensive review on the medicinally valuable endosymbiotic fungi *Penicillium chrysogenum*

**DOI:** 10.1007/s00203-023-03580-2

**Published:** 2023-05-17

**Authors:** Rawan Shaaban, Mohamed S. Elnaggar, Noha Khalil, Abdel Nasser B. Singab

**Affiliations:** 1 Department of Pharmacognosy and Medicinal Plants, Faculty of Pharmacy, Future University in Egypt, Cairo, 11835 Egypt; 2 Department of Pharmacognosy, Faculty of Pharmacy, Ain-Shams University, Cairo, 11566 Egypt; 3 Center of Drug Discovery Research and Development, Ain-Shams University, Cairo, 11566 Egypt

**Keywords:** Endosymbiotic fungus, *Penicillium chrysogenum*, Secondary metabolites, Cytotoxicity, Antimicrobial

## Abstract

Recently, it has been shown that metabolites derived from endosymbiotic fungi attracted high attention, since plenty of them have promising pharmaceutical applications. The variation of metabolic pathways in fungi is considered an optimistic source for lead compounds. Among these classes are terpenoids, alkaloids, polyketides, and steroids, which have proved several pharmacological activities, including antitumor, antimicrobial, anti-inflammatory, and antiviral actions. This review concludes the major isolated compounds from different strains of *Penicillium chrysogenum* during the period 2013–2023, together with their reported pharmacological activities. From literature surveys, 277 compounds have been identified from *P. chrysogenum,* which has been isolated as an endosymbiotic fungus from different host organisms, with specific attention paid to those showing marked biological activities that could be useful in the pharmaceutical industry in the future. This review represents documentation for a valuable reference for promising pharmaceutical applications or further needed studies on *P. chrysogenum.*

## Introduction

The genus *Penicillium* includes four subgenera*: Penicillium, Furcatum, Biverticillium, and Aspergilloides. Penicillium* is the subgenus that has been examined the most from the genus of *Penicillium* fungi (Kumar et al. 2018).

It is an anamorphic ascomycete with over 354 species (Nielsen et al. 2017) and is also known for its diverse spectrum of bioactive secondary metabolites with potential pharmacological actions, including antifungal, antibacterial, immunosuppressant and cholesterol-lowering agents (Rabha and Jha 2018). Studies on *Penicillium* have shown that the species taxonomical classification idea, based not only on DNA sequences, but also on ecological, morphological, and exo-metabolome profiles, provides a more accurate and true classification (Barreto et al. 2011).

**Fig. 1:**
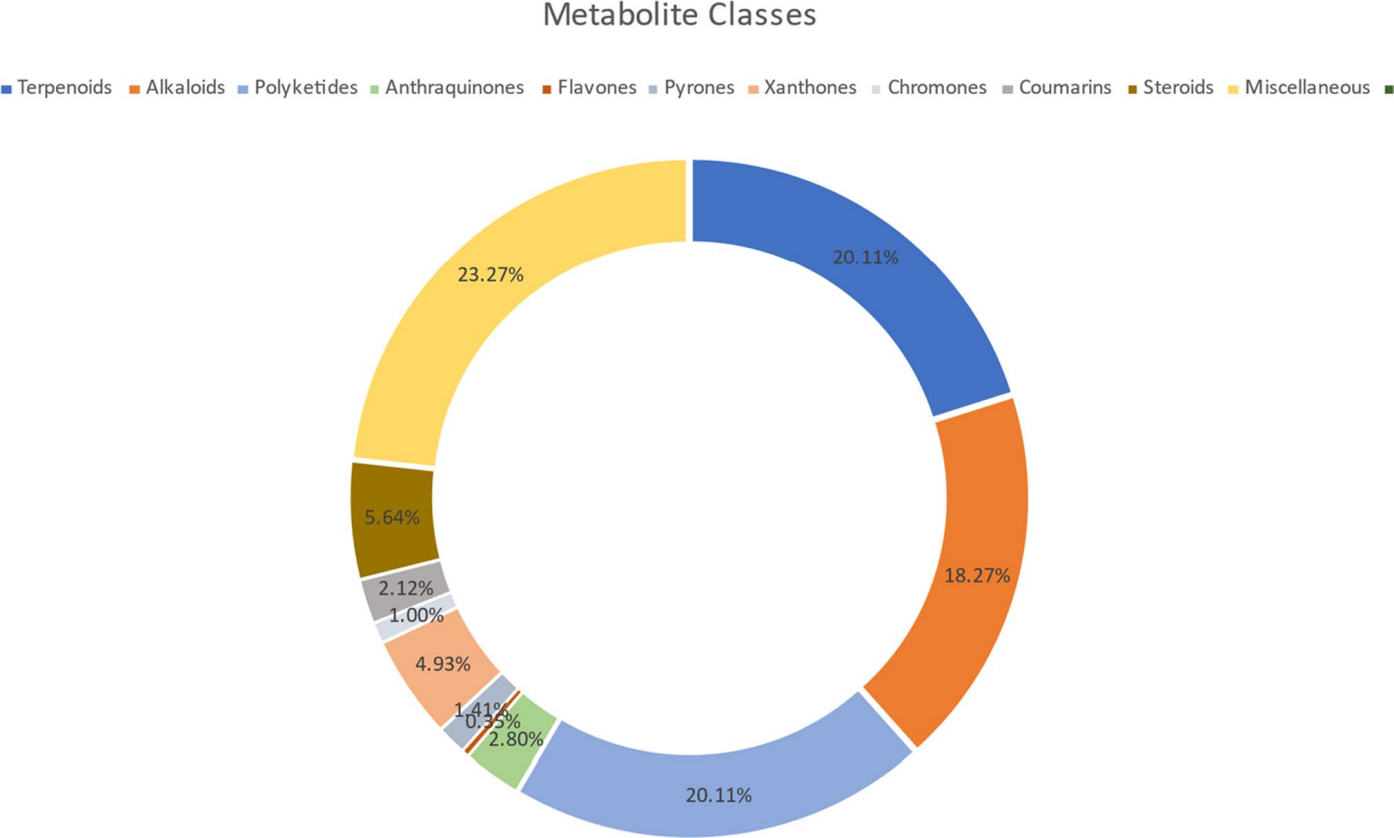
Percentage of classes of secondary metabolites identified in *P. chrysogenum*

**Fig. 2:**
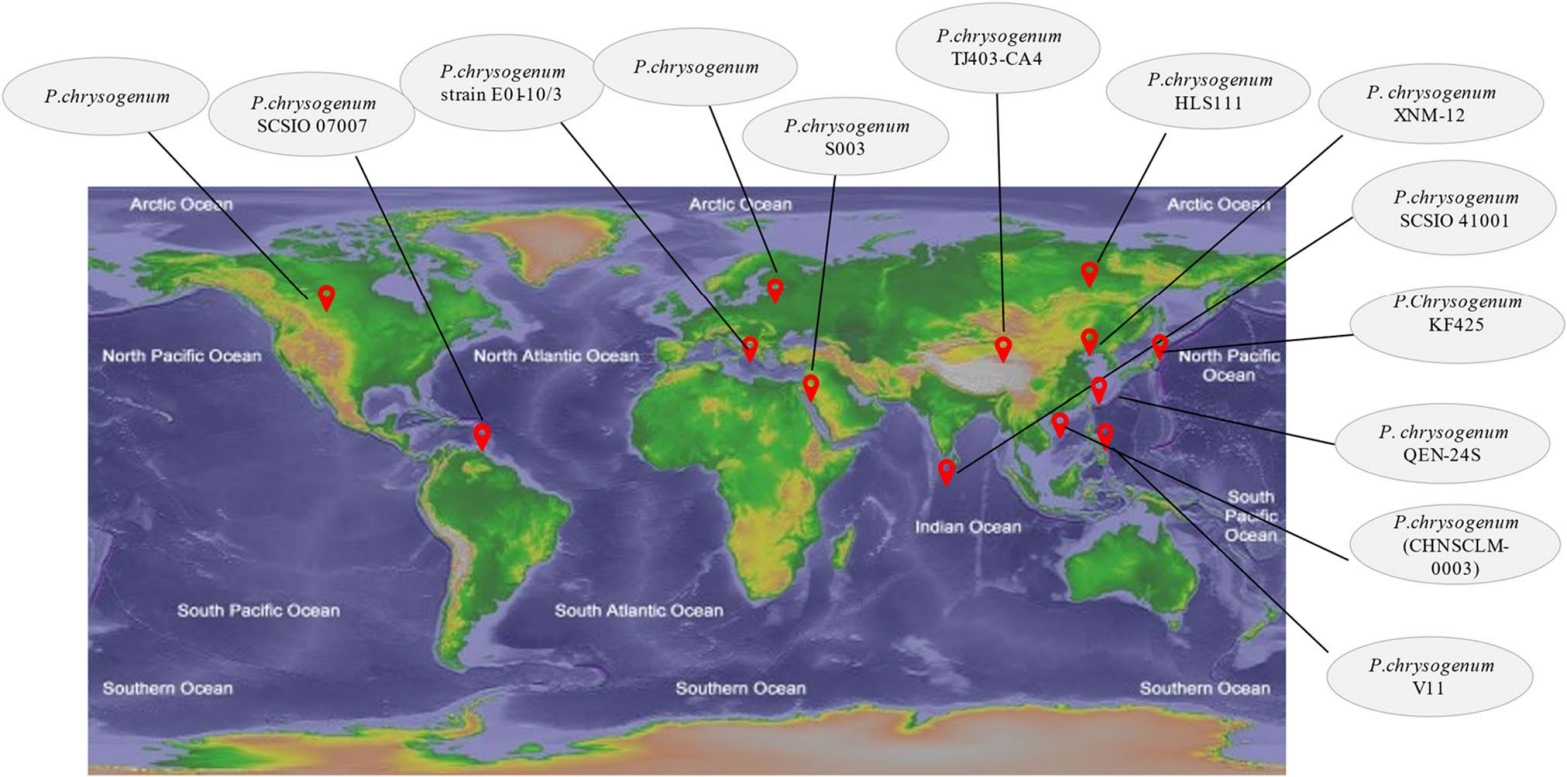
A map displaying the source of *P. chrysogenum* reviewed

**Fig. 3:**
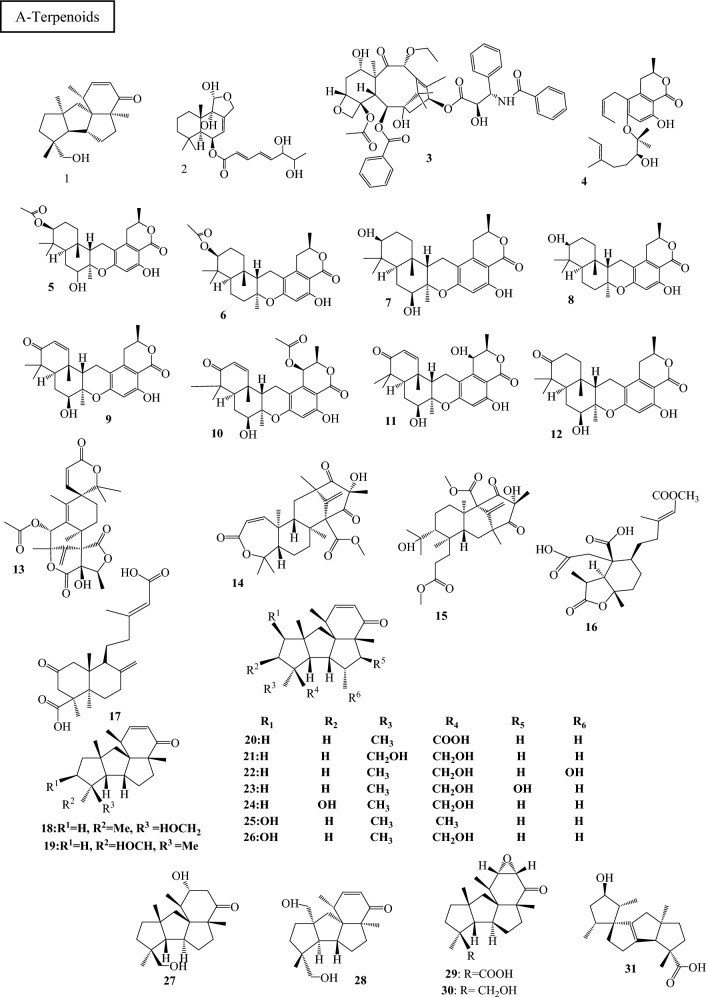

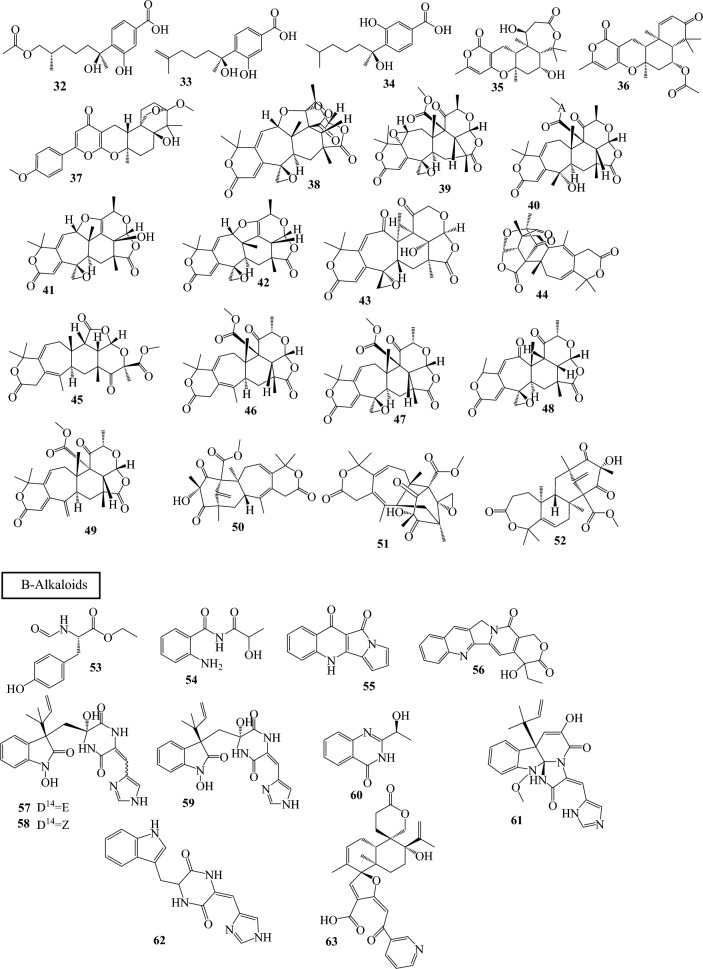

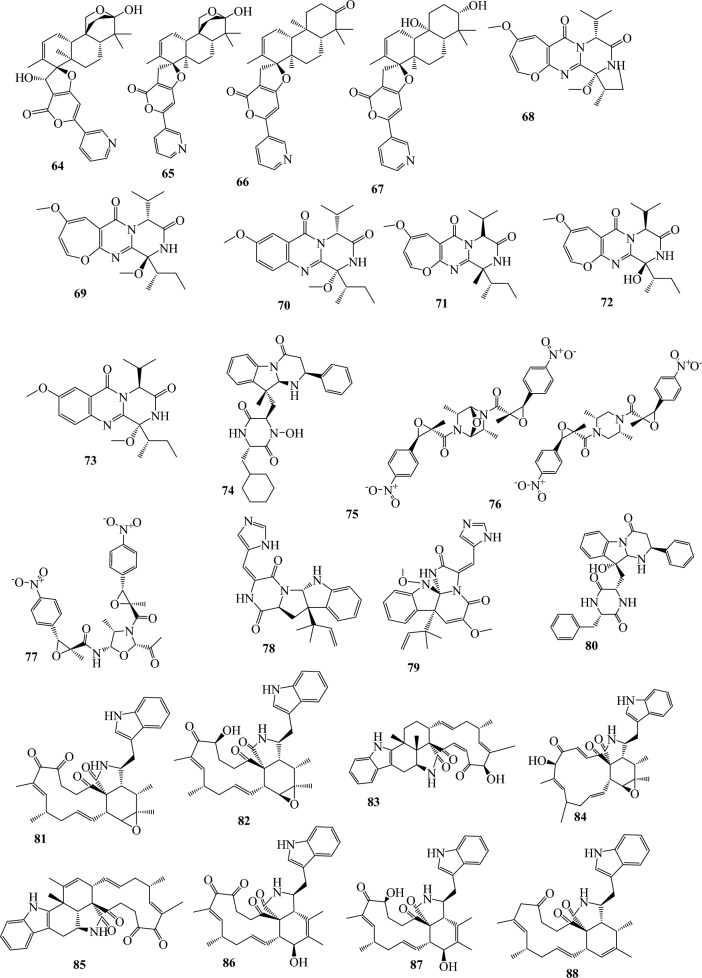

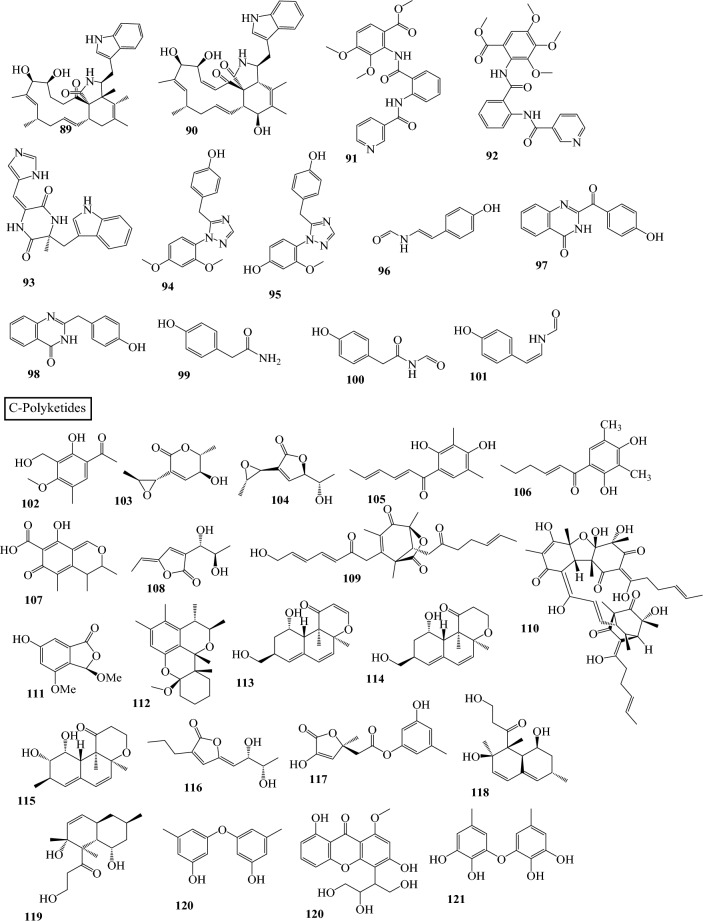

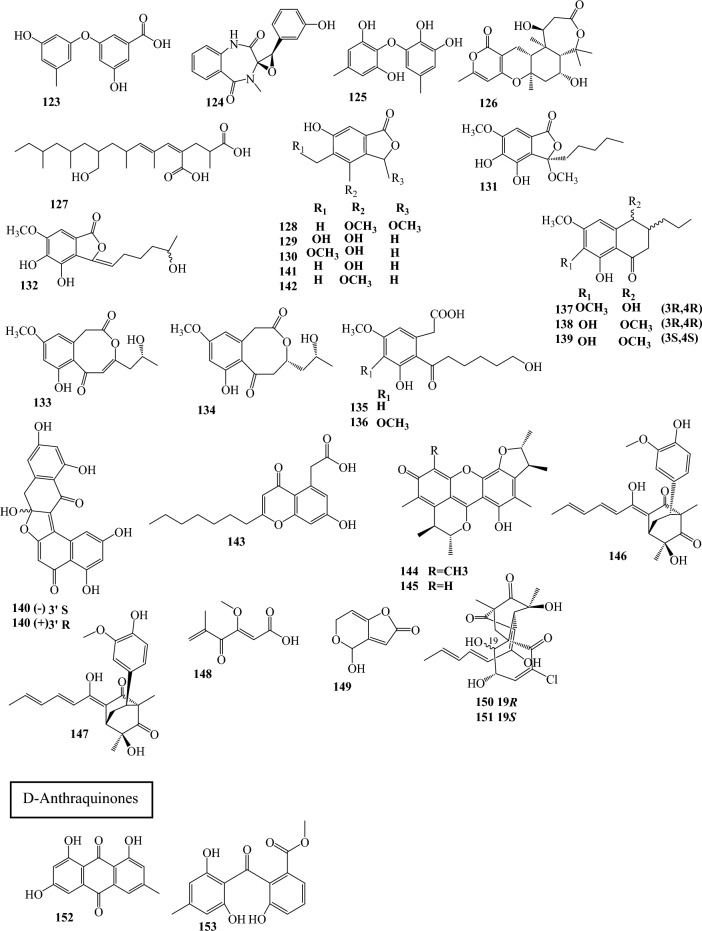

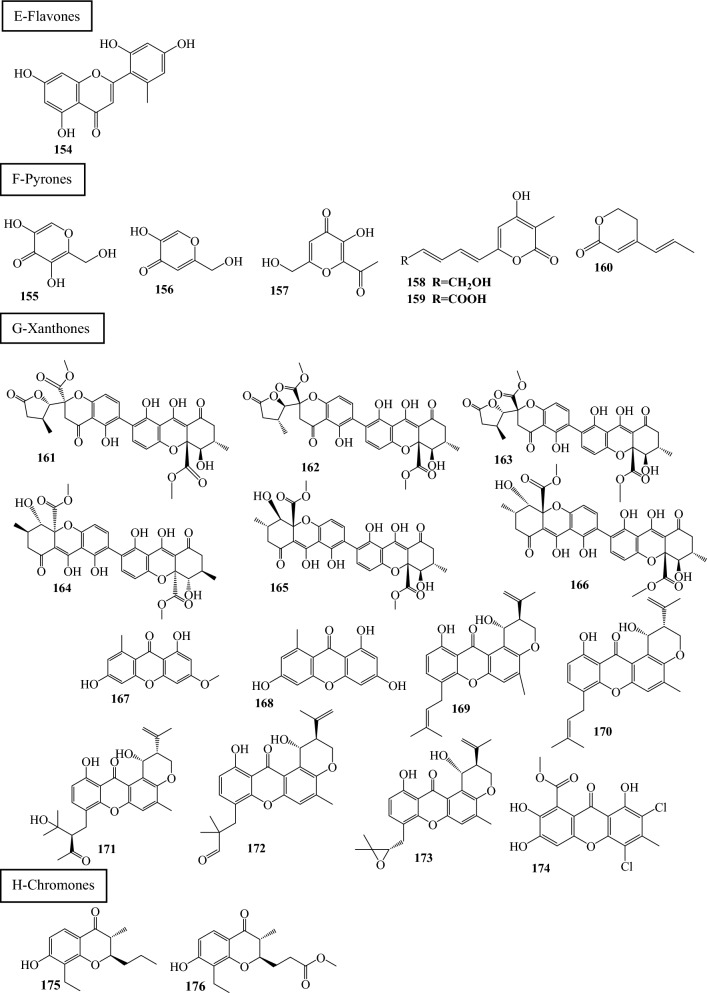

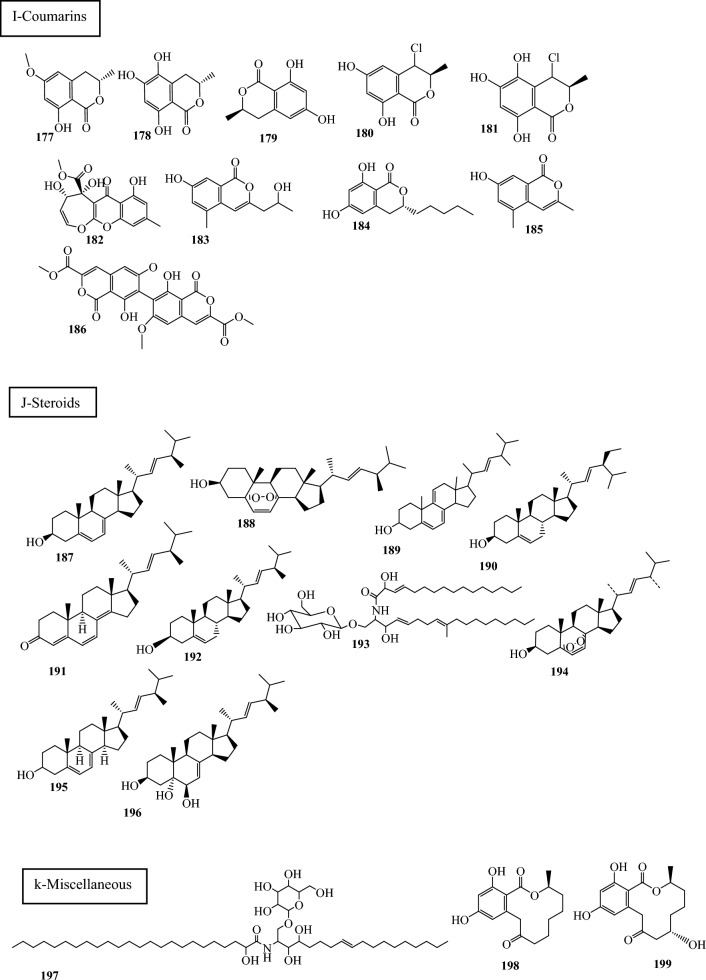

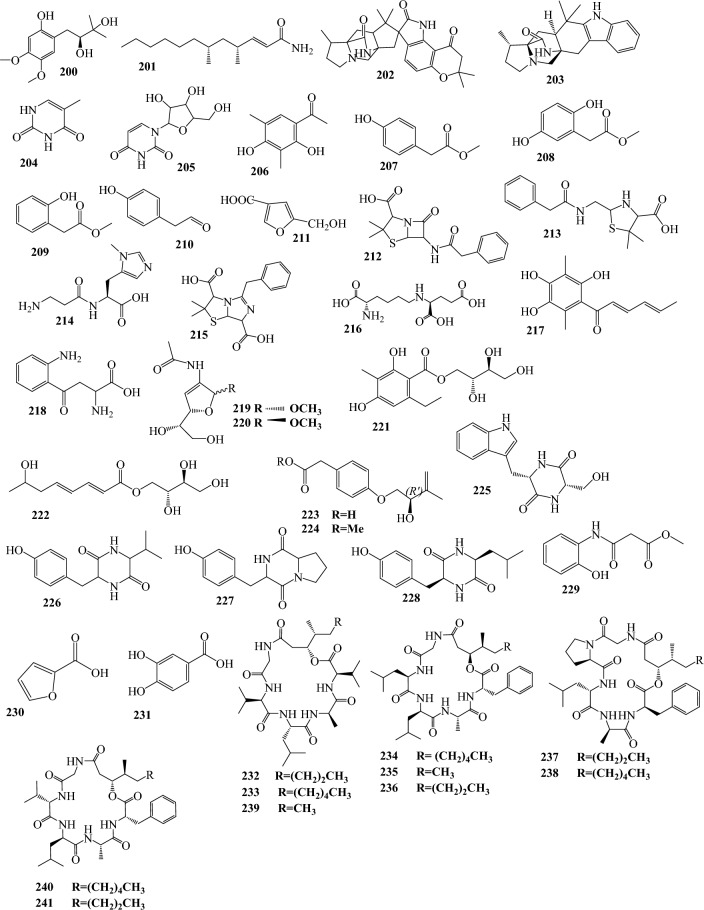

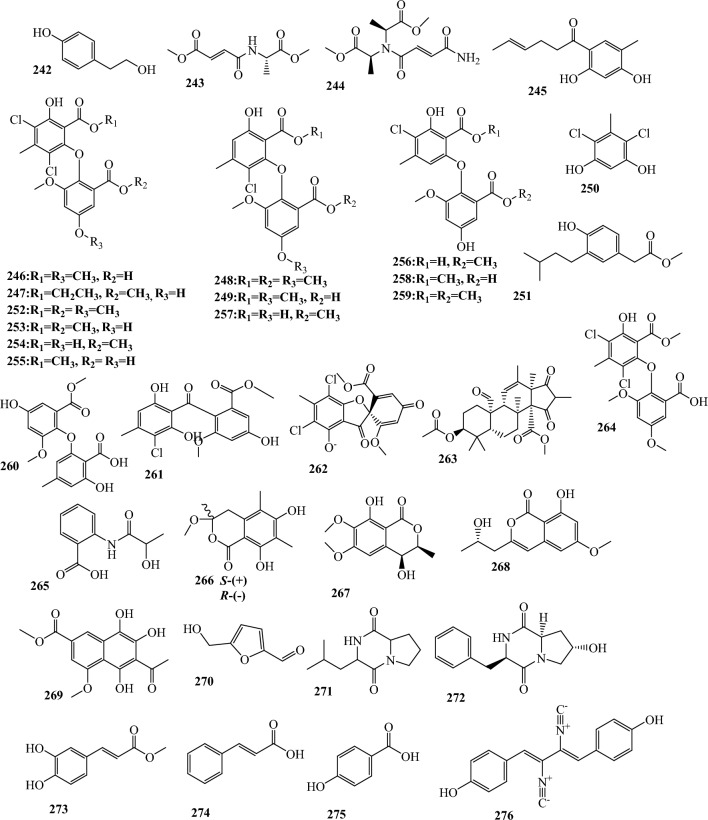

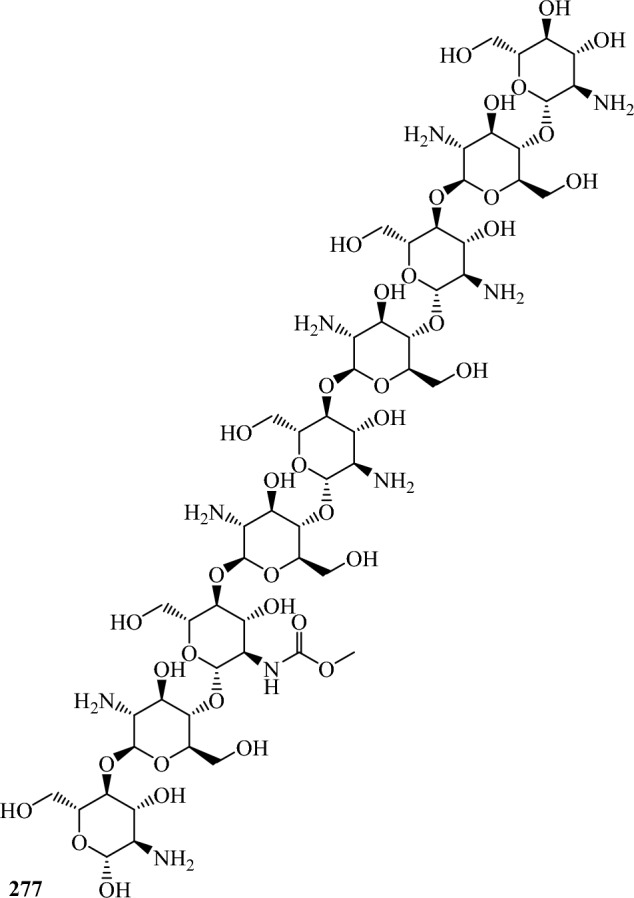
Structures of identified metabolites in *P. chrysogenum*

Marine-derived fungi from the *Penicillium* genus have gained attention as a beneficial source of new characteristic natural products with potential applications in industry, agriculture, and medicine (Kodoli et al. 2021). Marine *Penicillium* fungi have been found in sediments, mangroves, sponges, and algae, and have been shown to have high novelty for more than 390 new metabolites in the last decade, including alkaloids, polyketides, terpenes, and macrolides (Yang et al. 2021) that possess important biological activities such as anticancer, antimicrobial, anti-inflammatory and larvicidal actions with prospective applications in new drug development (Elkhawas et al. 2020; Singab et al. 2022). The *Penicillium chrysogenum* species, which belongs to the *Penicillium* subgenus, is particularly interesting due to its ability to produce penicillin and small antifungal proteins, making it beneficial for controlling fungal infections formed by other filamentous fungi (Martín 2020). This review concentrates on *P. chrysogenum* bioactive metabolites and their biological properties through the years 2013–2023. Various online databases were utilized, such as Web of Science, Marinlit, and Scifinder. The purpose of this review is to highlight all the progress made during the previous decade concerning the potential application of the isolated biomolecules (277 compounds and miscellaneous as shown in Table 1) from different strains of *P. chrysogenum*. This review deals with the chemical nature along with the reported pharmacological activities of the stated secondary metabolites, in addition to several miscellaneous compounds (Figs. 1, 2 and 3).

### K-Miscellaneous

The antimicrobial proteins (AMPs) named PAF and PAFB were secreted from the filamentous fungus *P. chrysogenum* Q176 (Huber et al. 2020).

The *P. chrysogenum* Q176 antifungal protein C (PAFC) was also characterized, and was different phylogenetically from the two *Penicillium* AMPs, PAF and PAFB (Holzknecht et al. 2020).

A new ascomycete fungus X5 was identified as *P. chrysogenum* which had hyperproducer activity of a serine alkaline protease (SAPTEX) (9000 U/mL) (Benmrad et al. 2018).

An acetyl xylan esterase (PcAxe) was cloned from *P. chrysogenum* P33 and expressed in *Pichia pastoris* GS115. rPcAxe comprises a domain of carbohydrate esterase and 62 domains of a glycosyl hydrolase family (Yang et al. 2017).

Two RG I-degrading enzymes, termed endo-RG and exo-RG lyases were reported to be secreted by *P.* *chrysogenum* 31B. The enzyme precisely acts on rhamnose (Rha) at the non-reducing end of RG oligosaccharides, but had not shown any action on flavonoid glycosides (Matsumoto et al. 2017).

*P. chrysogenum* could produce β-glucanase enzyme (El-Shora et al. 2019).

The filamentous ascomycete *P. chrysogenum* culture supernatant abundantly secreted the cysteine-rich, cationic, antifungal protein PAF (Sonderegger et al. 2017).

Chitosan (277) was extracted from broth culture of *P. chrysogenum* (Alagesan et al. 2016).

Only asexual reproduction is known to be used by *P. chrysogenum*. But further proof points to the possibility of sexual reproduction with an unidentified sexual stage*. P. chrysogenum* has recently been found to contain the mating-type (MAT) and pheromone signaling genes, which are associated with mating in other sexual fungus. In heterothallic ascomycete fungi, complementing MAT1-1 and MAT1-2 isolates are required for sex to occur. In contrast to the original Fleming strain, which has the opposite MAT1-2 locus, NRRL1951 (*P. chrysogenum* derivatives) has a MAT1-1 locus with a MAT1-1–1 gene encoding a putative alpha-box transcription factor. Sexual crosses can be used to develop new strains with improved industrial characteristics (Böhm et al. 2013).

**Table 1:** Classes of secondary metabolites identified in different strains of *P. chrysogenum*

Compound no.	Host organism	*P.chrysogeum* strain	Compound name	References
A-Terpenoids				
1	Seawater sample from the Indian Ocean	*P. chrysogenum* Y19-1	Conidiogenone C	(Li et al. 2023)
2	Acquired from a marine red alga, *Grateloupia turuturu*, obtained from the shoreline zone of Qingdao, China	*P. chrysogenum* LD-201810	Chrysoride A	(Huang et al. 2022)
3	The Pacific yew tree’s bark (*Taxus brevifolia*)	*P. chrysogenum* R16	Taxol	(El-Sayed et al. 2020)
4	Unidentified marine organism	*P. chrysogenum* S-3–25	Chrysomutanin	(Qiao et al. 2020)
5			3-Acetyl chrodrimanin F	
6			3-Acetoxypentacecilide A	
7			Chrodrimanin F	
8			3-Hydroxypentacecilide	
9			Chrodrimanin E	
10			Chrodrimanin B	
11			Chrodrimanin A	
12			Chrodrimanin H	
13			Austin	
14			Preaustinoid A2	
15			(−)- Preaustinoid D	
16	*Huperzia serrata* plant	*P. chrysogenum* MT-12	Penicichrysogene A	(Qi et al. 2020)
17			Penicichrysogene B	
1*	The arthropod *Cryptotympana atrata*	*P. chrysogenum* TJ403-CA4	Conidiogenone C	(Zhang et al. 2020)
18			Conidiogenone D	
19			Conidiogenone F	
20			20β-Carboxyl conidiogenone C	
21			19α-Hydroxy conidiogenone C	
22			7α-Hydroxy conidiogenone C	
23			8β-Hydroxy conidiogenone C	
24			13β-Hydroxy conidiogenone C	
25			Conidiogenone E	
26			12β-Hydroxy conidiogenone C	
27			Conidiogenone I	
28			Conidiogenone J	
29			20β-Carboxyl conidiogenone K	
30			Conidiogenone K	
31			Spirograterpene A	
32	A marine alga’s solid culture	*P. chrysogenum* LD-201810	(7*S*,11*S*) −( +)-12-Acetoxysydonic acid	(Jiang et al. 2020)
33			(*S*)−( +)-11-Dehydrosydonic acid	
34			Sydonic acid	
35			Asperdemin	
36			Asperversin G	
37	A sediment sample collected from the Indian Ocean	*P. chrysogenum* SCSIO 41,001	Yaminterritrem C	(Chen et al. 2017)
38–45	The fermentation cultures of *Huperzia serrata*	*P. chrysogenum* MT-12	Chrysogenolides (A − H)	(Qi et al. 2017b)
46			Berkeleyacetal A	
47			Berkeleyacetal B	
48			Berkeleyacetal C	
49			Purpurogenolide C	
50			Berkeleydione	
51			22-Epoxyberkeleydione	
52			Berkeleyone B	
B-Alkaloids				
53	A seawater sample from the Indian Ocean	*P. chrysogenum* Y19-1	Ethyl formyltyrosinate	(Li et al. 2023)
54			N-(2-hydroxypropanoyl)-2-aminobenzoic acid amide	
55	From *Cliona sp.* From Red Sea, Egypt	*P. chrysogenum* EFBL	Quinolactacide	(Al-Saleem et al. 2022)
56			Camptothecin	
57	Sample of deep sea hydrothermal vent environment from the Western Atlantic	*P. chrysogenum* SCSIO 07,007	Penilline A	(Han et al. 2020)
58			Penilline B	
59			Penilline C	
60			Chrysogine	(Han et al. 2020) (Visagie et al. 2014) (Frisvad et al. 2004)
61			Meleagrin	
62			Penilloid A	(Han et al. 2020)
63	The marine brown alga *Leathesia nana* (Chordariaceae)	*P. chrysogenum* XNM-12	Oxalicine C	(Xu et al. 2020)
64			Decaturin B	
65			Decaturin C	
66			Decaturin D	
67			Decaturin F	
68	Fresh Gorgonian *Dichotella gemmacea* was collected from the South China Sea	*P. chrysogenum* (CHNSCLM‐0019)	Chrysopiperazine A	(Xu et al. 2019)
69			Chrysopiperazine B	
70			Chrysopiperazine C	
71			Versicoloid A	
72			Versicoloid B	
73			Versicomide C	
74	Unidentified marine in Red Sea	*P. chrysogenum*	Haenamindole	(Hawas and Abou El-Kassem 2019)
75–77	Indian Ocean deep-sea sediment	*P. chrysogenum* SCSIO 41,001	Chrysamides A-C	(Wang et al. 2018)
61*	*Gelliodes carnosa* marine sponge, Lingshui Bay, Hainan Province, China	*P. chrysogenum* HLS111	Meleagrin	(Zhen et al. 2018) (Frisvad et al. 2004)
78			Rcqueforcine C	
79			Oxaline	(Zhen et al. 2018)
80			Citreoindole	
74*			Haenamindole	
81	The vein of *Myoporum bontioides* (Siebold & Zucc.) A. Gray, collected from the mangrove in Leizhou Peninsula	*P. chrysogenum* V11	Chaetoglobosin C	(Zhu et al. 2018) (Zhu et al. 2017)
82			Chaetoglobosin F	
83			Penochalasin I	
84			Chaetoglobosin A	
85			Penochalasin K	
86			Chaetoglobosin G	(Huang et al. 2016)
87			Chaetoglobosin E	
88			Penochalasin J	
89			Armochaetoglobosin I	
90			Cytoglobosin C	
91	A sediment sample collected from the Indian Ocean	*P. chrysogenum* SCSIO 41,001	Terremide D	(Chen et al. 2017)
92			Methyl3,4,5-trimethoxy-2-(2-(nicotinamido)benzamido) benzoate	
93	Different olive tree organs collected from its local habitat in Siwa Oasis, Western desert, Egypt	*P. chrysogenum mycelia*	Dehydrohistidyltryptophenyl-diketopiperazine (DHTD)	(Mady et al. 2016)
61*			Meleagrin	
79*			Roquefortine C	
94	*Sargassum palladium*, a marine brown alga, was found in China's Fujian Province	*P. chrysogenum* EN118	Chrysotriazole A	(An et al. 2013)
95			Chrysotriazole B	
96			N-[(2E)-(4-Hydroxyphenyl)ethenyl]formamide	
97			2-(4-Hydroxybenzoyl)-4(3H)-quinazolinone	
98			2-(4-Hydroxybenzyl)quinazolin-4(3H)-one	
99			2-(4-Hydroxy-phenyl)acetylamide	
100			N-[2-(4-Hydroxyphenyl)acetyl]formamide	
101			N-[(2Z)-(4-Hydroxyphenyl)ethenyl]formamide	
C-Polyketides				
102	From the mangrove swamp's intertidal zone in the Pangkep district of Indonesia's South Sulawesi province	*P. chrysogenum* ZZ1151	Communol G	(Newaz et al. 2022)
103	From *Cliona sp.* From Red Sea, Egypt	*P. chrysogenum* EFBL	Aspyrone	(Al-Saleem et al. 2022)
104			Asperlactone	
105			Sorbicillin	
106			Dihydrosorbicillin	
107			Citrinin	
108	The *Grateloupia turuturu* red alga marine collected from Qingdao, China	*P.* *chrysogenum* LD-201810	Penilactonol A	(Jiang et al. 2020)
109	The marine sponge *Theonella swinhoei* collected from the Xisha Islands, South China Sea	*P. chrysogenum* 581F1	13-Hydroxy-dihydrotrichodermolide	(Cao et al. 2020)
110			10,11,27,28-Tetrahydrotrisorbicillinone C	
111	The internal tissue of the marine red alga *Grateloupia turuturu*	*P. chrysogenum* AD-1540	Penicichrysogenins	(Zhao et al. 2018)
112			Penicitols	
113–115	The deep-sea sediment of the South Atlantic Ocean	*P. chrysogenum* MCCC 3A00292	Peniciversiols A − C	(Niu et al. 2019)
116–117			Penicilactones A-B	
118			Decumbenone A	
119			Decumbenone B	
120			3,3′-Dihydroxy-5,5′-dimethyldiphenyl ether	
121			3,8-Dihydroxy-4-(2,3-dihydroxy-1- hydroxymethylpropyl)-1-methoxyxanthone	
122			Aspermutarubrol	
123			3-Hydroxy-5-(3-hydroxy-5-methylphenoxy)benzoic acid	
124			Cyclopenol	
125			Violaceol-II	
126			Asperdemin	
127			Radiclonic acid	
128–139	*Huperzia serrata* (Thunb. ex-Murray) Trev. collected from Nanping, Fujian Province, China	*P. chrysogenum* MT-12	Penicichrysogenins A-L	(Qi et al. 2017a)
140			Asperlone A	
141			4,6-Dihydroxy-5- methylphthalide	
142			6-Hydroxy-4-methoxy-5-methylphthalide	
143			5-Carboxymethyl-2-heptyl-7-hydroxychromone	
144	A sediment sample collected from the Indian Ocean	*P. chrysogenum* SCSIO 41,001	Penicitrinone F	(Chen et al. 2017)
145			Penicitrinone A	
146–147	Sediments collected from the South China Sea	*P.chrysogenum* PJX-17	Sorbicatechols A and B	(Peng et al. 2014)
148	The trunk of *Strychnos toxifera,* collected from Manaus, Brazil	*P. chrysogenum*	Penicillic acid	(Koolen et al. 2014)
149			Patulin	
150–151	Unidentified	*P. chrysogenum*	Chloctanspirone A and B	(Bladt et al. 2013)
D-Anthraquinones				
152	*Eucommia ulmoides* Oliver’s healthy bark obtained from Kunming Botanical Garden, Yunnan, China	*P. chrysogenum* CF0105	Emodin	(Liu et al. 2020)
153			Moniliphenone	
E-Flavones				
154	A gorgonian *Carijoa sp*. collected from the South China Sea	*P. chrysogenum*	Penimethavone A	(Hou et al. 2016)
F-Pyrones				
155	From *Cliona sp.* From Red Sea, Egypt	*P. chrysogenum* EFBL	3-Hydroxy Kojic acid	(Al-Saleem et al. 2022)
156			Kojic acid	
157			Acetyl Kojic acid	
158–159	A deep-sea hydrothermal vent environment sample collected from the Western Atlantic Ocean	*P. chrysogenum* SCSIO 07,007	Chrysopyrones A and B	(Han et al. 2020)
160	A sediment sample collected from the Indian Ocean	*P. chrysogenum* SCSIO 41,001	(E)-4-(propen-1-yl)-5,6-dihydro-2H-pyran-2-one	(Chen et al. 2017)
G-Xanthones				
161–163	From Lingshui Bay *Gelliodes carnosa* sponge from Hainan Province, China	*P. chrysogenum* HLS111	Chrysoxanthones A-C	(Zhen et al. 2018)
164			Secalonic acid A	
165			Secalonic acid D	
166			Secalonic acid F	
167			Griseoxanthone C	
168			Norlichexanthone	
161–162*	Marine red alga *Grateloupia turuturu*	*P. chrysogenum* AD-1540	Chrysoxanthones A and B	(Zhao et al. 2018)
169			Shamixanthone	
170			Epishamixanthone	
171			Aspergixanthone	
172			Ruguloxanthone	
173			Tajixanthone	
174	A deep-sea sediment from the Indian Ocean	*P. chrysogenum* SCSIO 41,001	Chrysoxanthone	(Wang et al. 2018)
H-Chromones				
175–176	The healthy bark of *Eucommia ulmoides* Oliver obtained from Kunming Botanical Garden, Yunnan, China	*P. chrysogenum* CF0105	Penicichromanone A and B	(Liu et al. 2020)
I-Coumarins				
177	A sediment of Wadi Lajab, located 15 km northwest of Jazan, KSA	*P. chrysogenum (GenBank accession No. MH127462)*	6-Methoxy mellein	(Orfali et al. 2022)
178			5,6-Dihydroxymellein	
179			6-Hydroxymellein	
180			4-Chloro-6-hydroxymellein	
181			4-Chloro-5,6-di-hydroxymellein	
182	The healthy bark of *Eucommia ulmoides* Oliver obtained from Kunming Botanical Garden, Yunnan, China	*P. chrysogenum* CF0105	Conioxepinol C	(Liu et al. 2020)
183	*Gelliodes carnosa* marine sponge collected from Lingshui Bay, Hainan Province, China	*P. chrysogenum* HLS111	7-Hydroxy-3-(2-hydroxypropyl)-5-methyl-isochromen-1-one	(Zhen et al. 2018)
184			Penicisimpin B	
185			7-Hydroxy-3,5-dimethyl-isochromen-1-one	
186	Sediment sample collected from the Indian Ocean	*P. chrysogenum* SCSIO 41,001	Bipenicilisorin	(Chen et al. 2017)
J-Steroids				
187	Collected from the seawater sample of the Indian Ocean	*P. chrysogenum* Y19-1	Ergosterol	(Li et al. 2023)
188			Ergosterol peroxide	
189	From *Cliona sp.* From Red Sea, Egypt	*P. chrysogenum* EFBL	Dehydroergosterol	(Al-Saleem et al. 2022)
190			Stigmasterol	
191			Ergosta-4,6,8(14),22-tetraen-3-one	
192			Brassicasterol	
193	Deep-sea sediment from the Red Sea	*P. chrysogenum* S003	LAMA	(Alshehri et al. 2020)
187*			Ergosterol	
194			Epidioxyergosterol	
195	A sample from the North China Sea	*P. chrysogenum*	(22E,24R)-ergosta-5,7,22-triene-3β-ol	(Wang et al. 2014)
187*	The trunk of *S. toxifera,* collected from Manaus, Brazil	*P. chrysogenum*	Ergosterol	(Koolen et al. 2014)
192*			Brassicasterol	
188*			Ergosterol peroxide	
196			Cerevisterol	
K-Miscellaneous				
197	Collected from the seawater sample of the Indian Ocean	*P. chrysogenum* Y19-1	Cerebroside A	(Li et al. 2023)
198			Dihydroresorcylide	
199			7-Hydroxydihydroresorcylide	
200	Collected from intertidal zone of mangrove swamp of Pangkep district of South Sulawesi province, Indonesia	*P. chrysogenum* ZZ1151	Peniprenylphenol A	(Newaz et al. 2022)
201			Penicimumide	
202			Peniciherquamide A	
203			Preparaherquamide	
204			Thymine	
205			Uridine	
206			Clavatol	
207			4-Hydroxybenzeneacetic acid methyl ester	
208			2,5-Dihydroxyphenylacetic acid methyl ester	
209			2-Hydroxyphenylacetic acid methyl ester	
210			4-Hydroxyphenylethanone	
211	From *Cliona sp.* From Red Sea, Egypt	*P. chrysogenum* EFBL	Fulfuran	(Al-Saleem et al. 2022)
212			Penicillin G	
213			Penilloic acid	
214			Anserine	
215			Penillic acid	
216			L-saccharopine	
217			Sohirnone B	
218			kynurenine	
212*	Unidentified	*P. chrysogenum* KF425	Penicillin G	(Horii et al. 2020)
219–220	Obtained from the inner tissue of marine red alga *Grateloupia turuturu*	*P. chrysogenum*	Penichryfurans A and B	(Chen et al. 2022)
221–222	The marine brown alga *Leathesia nana* (Chordariaceae), collected from Weihai, Shandong Province, China	*P. chrysogenum* XNM-12	Penicierythritols A and B	(Xu et al. 2020)
223	The marine red alga *Grateloupia turuturu* collected from Qingdao, China	*P.* *chrysogenum* LD-201810	(2'R)-stachyline B	(Jiang et al. 2020)
224			(2'R)-westerdijkin A	
225	Deep-sea hydrothermal vent environment sample collected from the Western Atlantic Ocean	*P. chrysogenum* SCSIO 07,007	Cyclo-(Trp-Ser)	(Han et al. 2020)
226			Cyclo-(Val-Tyr)	
227			Cyclo (Tyr-Pro)	
228			Cyclo-(Leu-Tyr)	
229			2-(N-(2-hydroxyphenyl) carbamoyl)acetate	
230			2- furoic acid	
231			3,4-dihydroxybenzoic acid	
232–238	Gorgonian coral *Carijoa sp*. (GX-WZ-2010001) collected from Weizhou coral reefs, South China Sea	*P. chrysogenum* (CHNSCLM-0003)	Chrysogeamides A–G	(Hou et al. 2019)
239			Nodupetide	
240–241			Scopularides (A-B)	
242	Sediments nearby the East Sea, collected in Taiwan Strait, China	*P. chrysogenum* DXY-1	Tyrosol	(Chang et al. 2019)
243	*Myoporum bontioides* (Siebold & Zucc.) A. Gray collected from Leizhou Peninsula, China	*P. chrysogenum* V11	N-fumaryl-L-alanine dimethyl ester	(Zhu et al. 2018)
244			N,N-bis[(S)-1-methoxycarbonylethyl]fumaric diamide	
245			Sohirnone A	
246–249	Deep-sea sediment of the Indian Ocean	*P. chrysogenum* SCSIO 41,001	Chrysines A–D	(Wang et al. 2018)
250			Dichloroorcinol	
251			3-isopentyl-4-hydroxy phenylacetic acid methyl ester	
252			Methyl 3′- methoxy-3,5-dichloroasterric acid	
253			Methyl dichloroasterrate	
254			2,4-Dichloroasterric acid	
255			Geodin hydrate	
256			Methyl chloroasterrate	
257			5-Chloroasterric acid	
258			Iizukine A	
259			Penicillither	
260			Asterric acid	
261			Mono-chlorosulochrin	
262			( +)-Geodin	
263	*Gelliodes carnosa* marine sponge obtained from Lingshui Bay, Hainan Province, China	*P. chrysogenum* HLS111	Andrastins A (keto form and enol form)	(Zhen et al. 2018) (Visagie et al. 2014)
264	Marine red alga *Grateloupia turuturu*	*P. chrysogenum* AD-1540	Chrysines	(Zhao et al. 2018)
265	Unidentified marine	*P. chrysogenum*	2-(2-Hydroxypropanamido) benzoic acid (HPABA)	(Zhang et al. 2017)
266	Sediment sample collected from the Indian Ocean	*P. chrysogenum* SCSIO 41,001	( ±)-stoloniferol A	(Chen et al. 2017)
267			4-hydroxykigelin	
268			Diaporthin	
269			Methyl 6-acetyl-4-methoxy-5,7,8-trihydroxynaphthalene-2-carboxylate	
270	Mud sample separated from Lianyungang Sea, China	*P. chrysogenum* HGQ6	5-Hydroxymethyl-2-furancarboxaldehyde	(Guo et al. 2016)
265*	A sample from the North China Sea	*P. chrysogenum*	2-(2-hydroxypropanamido) benzoic acid	(Wang et al. 2014)
271			Cyclo-(Pro-Leu)	
272			Cyclo-(4-hydroxyl-Pro-Phe)	
273	Sediments collected in the South China Sea	*P. chrysogenum* PJX-17	Caffeic acid methyl ester	(Peng et al. 2014)
274	The trunk of *S. toxifera,* collected from Manaus, Brazil	*P. chrysogenum*	Cinnamic acid	(Koolen et al. 2014)
275			P-hydroxybenzoic acid	
276	Unidentified	*P. chrysogenum*	Xanthocillin X	(Bladt et al. 2013)

All structures were identified using extensive spectroscopical techniques such as UV, 1D and 2D. NMR and HRESIMS spectra, and literature data were used to determine their structures and absolute configurations

Compounds isolated and identified from *P. chrysogenum* in different host organisms

(*) repeated Compounds

### Pharmacological activities

#### Anti-fungal

According to Al-Saleem et al. (2022), *P. chrysogenum* extract exhibited significant antifungal activity towards *Candida albicans* and *Cryptococcus neoformans* with MIC 93.75 ± 0.55 and 19.53 ± 0.48 µg/mL, respectively. Moreover, kojic acid (156) revealed the same potency towards *Fusarium oxysporum* and *Cryptococcus neoformans* with MIC 39.06 ± 0.85 and 39.06 ± 0.98 µg/mL, respectively.

Holzknecht et al. (2020) reported that the antifungal protein C (PAFC) produced by *P. chrysogenum* Q176 was produced together with PAF and PAFB into the culture broth. Recombinant PAFC's functional characterization revealed a promising novel molecule for anti-*Candida* therapy. In pre-established biofilms of two strains of *C. albicans*, the planktonic cells were killed by the thermotolerant PAFC while the sessile cells’ metabolic activity decreased. One of the strains was a fluconazole-resistant that displayed greater PAFC sensitivity than the fluconazole-sensitive one. The absence of hemolytic activity supports the further use of PAFC in clinical therapy.

Huber et al. (2020) found that PAF and PAFB, the antimicrobial proteins (AMPs) secreted by the filamentous fungus *P. chrysogenum* Q176, are highly stable due to a compact disulfide-bond, β-fold structure. In micromolar doses, these two AMPs effectively prevented the growth of several fungi including: *Aspergillus fumigatus*, *Trichophyton spp., Aspergillus niger,* and *Candida spp.,* along with the *Neurospora crassa* and *Saccharomyces cerevisiae.*, which were vulnerable to both proteins since their growth diminished at 0.25–4 μM PAF or PAFB doses, respectively.

Xu et al. (2020) reported that penicierythritol A (221) isolated from endophytic *P. chrysogenum* XNM-12, the marine algal-derived fungus, had a moderate antifungal potential towards the plant pathogenic fungus *Alternaria alternata* with MIC 8 μg/mL.

As elaborated by Sonderegger et al. (2017), PAF, a cysteine-rich, cationic antifungal protein that is mostly made up of 55 amino acids, was abundantly generated by the filamentous ascomycete *P. chrysogenum*. *Botrytis cinerea* and *Aspergillus fumigatus*, two opportunistic human and plant diseases, were discovered to be particularly inhibited, even though in vitro and in vivo tests had shown that they were inert against mammalian cells.

Zhu et al. (2017) have identified penochalasin K (85) from the mangrove endophytic fungus *P. chrysogenum* V11 culture. In fact, it exhibited serious inhibitory actions towards *Colletotrichum gloeosporioides* and *Rhizoctonia solani* (MICs Values = 6.13, 12.26 μM, respectively), which were notably higher than carbendazim.

Previous study by Huang et al. (2016) showed that chaetoglobosin A (84), chaetoglobosin C (81), chaetoglobosin E (87) and armochaetoglobosin I (89) were isolated from the culture of *P. chrysogenum* V11. They remarkably inhibited the plant pathogenic *Rhizoctonia solani* fungus ((MICs) = 11.79–23.66 μM), while chaetoglobosin A (84), chaetoglobosin E (87) and penochalasin J (88), significantly inhibited *Colletotrichum gloeosporioides* (MICs Values = 23.58–47.35 μM), revealing a higher activity than carbendazim. The findings revealed that the corresponding isolates could be greatly employed as fungicides or as primes of newly fungicidal agents against the mentioned pathogenic fungi.

According to Lopes et al. (2013), *P. chrysogenum* IFL1 produced bioactive compounds that spread on agro-industrial residues, cheese whey, and grape trash. The cheese whey culture filtrate hindered the development of the fungus *Fusarium oxysporum* as well as the amoeba *Acanthamoeba polyphaga*.

### Anti-viral

In vitro as well as in vivo studies by Huber et al. (2020) on PAF and PAFB, the two antimicrobial proteins (AMPs) secreted by the filamentous fungus *P. chrysogenum* Q176, displayed that they had antiviral activity without triggering any cytotoxic effects or hemolytic activity on mammalian cells. Experiments in human cervix cancer cells showed that they both reduced Human Coronavirus cytopathogenic effects. Apparently, it was the very first study on the antiviral ability of small, cysteine-rich and cationic proteins derived from fungi.

Hawas and Abou El-Kassem (2019) used a scale-up fermentation approach that yielded haenamindole (74), an uncommon diketopiperazine (DKP) alkaloid, from the endophytic fungus *P. chrysogenum* in a biomaltpeptone medium. This step was proceeded by cytotoxicity-guided fractionation. It showed low HCV protease potential with an IC_50_ value of 76.3 μM.

Study by Peng et al. (2014) isolated sorbicatechol A and sorbicatechol B (146,147), from the deep-sea sediment-derived fungus *P. chrysogenum* strain PJX-17’s culture. Results revealed that both displayed activities against influenza virus A (H1N1), with IC_50_ at 85 and 113 μM, respectively.

### Anti-inflammatory

Liu et al. (2020) have isolated two novel chroman-4-ones named penicichromanone A (175) and penicichromanone B (176) along with three previously identified metabolites emodin (152), moniliphenone (153), and conioxepinol C (182), were attained from an endophytic fungus *P. chrysogenum*, separated from *Eucommia ulmoides* Oliver bark. The anti-inflammatory activity for all the obtained compounds were evaluated using HEK293 cells reporting that compounds (175), (152), (153) and (182) had powerful inhibitory actions on TNF-*α*-stimulated NF-κB activation.

According to Zhang et al. (2017), HPABA (265) is considered a potent anti-inflammatory compound obtained from the marine *P. chrysogenum* and was found to be an analogue of aspirin by structure.

In a previous study for Qi et al. (2017b), Compounds (40), (41), (43), (48), and (49) were obtained from the fermented cultures of a *Huperzia serrata* endophytic fungus, *P. chrysogenum* MT-12, demonstrated inhibition of the production of nitric oxide in the lipopolysaccharide-activated RAW 264.7 macrophage cytes with IC_50_ values range 4.3–78.2 μM (the standard, indomethacin, IC_50_ = 33.6 ± 1.4 μM)..

Wang et al. (2014) isolate*,* a new benzoic acid derivative, HPABA (265) from the fermented broth of *P. chrysogenum*., where it presented significant anti-inflammatory with pain killer activities when given at 100 mg/kg, while it showed no ulcerogenic actions.

### Cytotoxicity

Huang et al. (2022) work showed that chrysoride A (2) had moderate cytotoxicity towards HepG2 and HeLa cancer cell lines with IC_50_ values of 28.9 and 35.6 μM, respectively.

Chen et al. (2022) exposed compounds penichryfurans A and B (219–220) to Cell Counting Kit-8 (CCK-8) colorimetric assay towards A549, HepG2 and HeLa cell lines to evaluate their cytotoxic activity. Penichryfuran A (219) exhibited high cytotoxicity towards the HepG2 cell line with IC_50_ value of 9.0 μM.

According to Al-Saleem et al. (2022), cytotoxic activity towards the cell lines (HCT-1 and HEP-2) were investigated using MTT method. In HCT-116 colon carcinoma cells, the ethyl acetate extract of *P. chrysogenum* and Kojic acid (156) exhibited strong activity with IC_50_ 22.6 ± 0.8 and 23.4 ± 1.4 µg/mL, respectively, with respect to HEP-2 larynx carcinoma, the total extract and Kojic acid (156) had effective comparable cytotoxic profile against HEP-2 cell lines with same IC_50_ 30.8 ± 1.3 and 30.8 ± 1.2 µg/mL, respectively. These substantial antimicrobial and cytotoxic activities may be due to the presence of penicillin G (212), Kojic acid (156), sohirnone B (217), and camptothecin (56) as major constituents in the ethyl acetate extract of *P. chrysogenum*.

Regarding the quantitative methodology by TLC and HPLC, El-Sayed et al. (2020) revealed that *P. chrysogenum* is an effective taxol (3) producer. Antiproliferative behavior of taxol was tested towards several cell lines like liver cancer cells (HEPG2) and breast adenocarcinoma (MCF7) cell viability. A considerable impact was revealed (*p* value < 0.05), since both MCF7 and HEPG2 cell viability were declined particularly by increasing taxol concentrations. The isolated taxol (3) IC_50_ value was indicated around 3.3 and 3.7 µM against cell lines MCF7 and HEPG2, individually.

Jiang et al. (2020) investigated the cytotoxicity of the obtained compounds from the marine alga-derived *P. chrysogenum* strain LD-201810 fungus culture against six human cancer cell lines including A549, HeLa, THP-1, MCF-7, HepG2, and BT-549. (2’R)-westerdijkin A (224) demonstrated anticancer activity against the HepG2 cell line with an IC_50_ 22.0 μM. Furthermore, (S)-( +)-11-dehydrosydonic acid (33) showed significant activity towards THP-1 and A549 with an estimated IC_50_ values of 18.2 and 21.2 μM, respectively. All results suggested that (2’R)-westerdijkin A (224) and (S)-( +)-11-dehydrosydonic acid (33) had apoptosis-triggering action towards the HepG2, A549, and THP-1 cell lines, respectively.

Using MTT method, Qiao et al. (2020) evaluated the cytotoxic activity of the isolated metabolites from the diethyl sulphate (DES) mutant 3d10-01 of the marine-derived fungus *P. chrysogenum* S-3–25 on the human cancer cell lines, HeLa K562, BGC-823, HL-60, and A549. All the tested compounds revealed poor inhibitory action on the assayed cell lines, except chrysomutanin (4), 3-acetyl chrodrimanin F (5) and chrodrimanin F (7) which revealed higher inhibitory activities on HL-60 cells with an IC_50_ values 4.8, 8.1 and 8.7 μM, respectively.

Cao et al. (2020) used the surface plasmon resonance imaging (SPRi) method to reveal that 13-hydroxy-dihydrotrichodermolide (109) and 10,11,27,28-tetrahydrotrisorbicillinone C (110) had great affinity for eEF2K (cancer) with K_d_ values of 0.118 μM, 0.0746 μM for eEF2K, respectively.

In a previous study by Alshehri et al. (2020) stated that the chemical analysis of the *P. chrysogenum* S003 ethyl acetate extract yielded metabolites which were evaluated towards five human cancer cell lines by SRB assay. Ergosterol **(**187) and Epidioxyergosterol (194) showed promising cytotoxic activities against prostate (DU-145), hepatocellular (HepG2) cell lines, lung (A-549), and breast adenocarcinoma (MCF-7), with IC_50_ values of 1.50, 6.10; 2.89, 3.07 21.26, 19.3; and 16.95, 13.6; µM, respectively. However, LAMA **(**193) and Kojic acid **(**156) exhibited poor cytotoxic actions against all the corresponding cell lines.

According to Niu et al. (2019), the cytotoxic effects of all the identified compounds from *P. chrysogenum* MCCC 3A00292 the deep-sea-derived fungus solid cultures were tested for five human cancer cell lines: BEL-7402, BIU-87, ECA109, Hela-S3 and PANC-1. Peniciversiol A (113) showed a marked inhibitory potentials towards the BIU-87 cells (IC_50_ value of 10.21 μM), while metabolites Penicilactones A-B (116–117) and Decumbenone A-B (11–119) as well Aspermutarubrol (122), 3-hydroxy-5-(3-hydroxy-5-methylphenoxy)benzoic acid (123), Cyclopenol (124), Violaceol-II (125) had inhibitory effects towards the BEL-7402, BIU-87, and ECA109 cancer cell lines with an estimated IC_50_ values range from 7.70 to > 20 μM.

Hawas and Abou El-Kassem (2019) used a scale-up fermentation approach that yielded Haenamindole (74), an uncommon diketopiperazine (DKP) alkaloid of the endophytic fungus *P. chrysogenum* in biomaltpeptone media. This step was proceeded by cytotoxicity-guided fractionation within a group of up to 12 cancer cell lines. Results revealed substantial cytotoxicity of the metabolite, with definite selectivity for colon-38 carcinoma cells compared to human normal cells.

Ory et al. (2019) stated that the marine-derived fungus, *P. chrysogenum* MMS5 extract, shows strong antiproliferative action on breast cancer cells (MCF-7 cell line) in a real-case investigation. Its antiproliferative activity was validated with an IC_50_ of 0.10 μM on MCF-7 cells mainly due to the presence of high amounts of ergosterol.

A study by Zhen et al. (2018) showed that chrysoxanthones A–C (161–163) were obtained from the *P. chrysogenum* strain HLS111. In vitro cytotoxic activity against different human cell lines were estimated for these metabolites against renal carcinoma (A498), multiform glioblastoma (U87 MG), leukemia (HL60), non-small cell lung tumor (NCI-H1650), and colonic carcinoma (HT29), by the MTT method. The anticancer activities of compounds (161–163) diminished significantly compared to secalonic acid D which was previously isolated. The cytotoxic effect was reduced due to the presence of β-methyl-γ-lactone ring, meaning that the tricyclic nucleus was important to achieve bioactivity.

Chromatographic analysis performed by Zhao et al. (2018) on extracts of *P. chrysogenum* AD-1540, a marine algal-derived endophytic fungus which was separated from the red alga *Grateloupia turuturu* ‘s inner tissue, afforded chryxanthones A and B (161–162), two novel benzophenone derivatives. Their cytotoxic profile was evaluated towards six human cancer cell lines: MCF-7, A549, HeLa, BT-549, HepG2 and THP-1. Chryxanthone A (161) exhibited moderate activity towards BT-549 and HeLa cancer cell lines, with IC_50_ values of 20.4 and 23.5 µM, respectively, whereas chryxanthone B (162) inhibited the growth of A549 cell line selectively with an IC_50_ value of 20.4 µM.

The antimetastatic and antiangiogenic properties of the halotolerant fungus *P. chrysogenum-1 hPc-1* isolated from Tuz Lake Turkey, were assessed by Dikmen et al. (2017). Its extract activity on human umbilical vein endothelial cells (HUVEC) and colorectal cancer cells (Caco-2) was assessed using the WST-1 technique and real-time cell analysis system-DP. According to the results, mRNA expression levels of the genes for VEGF A, VEGF B, COX-10, EGFR, ANGPT-1, and IL-8 were lower in HUVEC and Caco-2 cells compared to the standard.

According to Zhu et al. (2017), MTT technique revealed the cytotoxicity profile of the isolated metabolites from the mangrove endophytic fungus *P. chrysogenum* V11 against three diverse human cancer cell lines, lung adenocarcinoma epithelial cell line (A549), gastric cancer cell line (SGC-7901), and a breast cancer cell line (MDA-MB-435). Penochalasin K (85) showed significant broad-spectrum inhibitory actions towards all the assayed cell lines (IC_50_ < 10 μM). Also, chaetoglobosin A (84), chaetoglobosin C (81) and penochalasin I (83) exhibited mild to noticeable inhibitory activities on the mentioned cell lines with IC_50_ values range of 6.56–37.56 μM.

Chen et al. (2017) revealed that bipenicilisorin (186), which was obtained from a marine-derived fungus *P. chrysogenum* SCSIO 41,001, displayed cytotoxic effects towards Huh-7, K562, and A549 cancer cell lines significantly with IC_50_ values at 2.59, 6.78, and 6.94 μM, respectively, while penicitrinone F (144) displayed a modest inhibitory effect against EV71 with IC_50_ 14.50 μM.

Huang et al. (2016) tested compounds obtained from the culture of *P. chrysogenum* V11 for their anticancer activity. Penochalasin I (83) showed significant activity against SGC-7901 and MDA-MB-435 cells (IC_50_ < 10 μM), while cytoglobosin C (90) had high activity against A549 and SGC-7901 cells (IC_50_ < 10 μM).

Guo et al. (2016) stated that 5-Hydroxymethyl-2-furancarboxaldehyde (270) recovered from the ethyl acetate extract of *P. chrysogenum* HGQ6 fermentation broth had activity towards BGC823 cell with the IC_50_ value of 0.19 mg/mL.

Previous studies by Hou et al. (2016) on *P. chrysogenum,* grown from a Gorgonian *Carijoa sp*. found in the South China Sea, yielded a novel flavone penimethavone A (154), with a unique methyl group at ring-B which is rarely found. It was tested in vitro for cytotoxicity against cervical cancer (HeLa), rhabdomyosarcoma, non-small cell lung cancer (A549) and human laryngeal epithelial (Hep-2) cell lines. Results indicated that it had moderate selective cytotoxic activity against (HeLa) and rhabdomyosarcoma cell lines, with IC_50_ values of 8.41 and 8.18 µΜ, respectively.

Mady et al. (2016) yielded meleagrin (61), by bioguided chromatographic analysis of the dichloromethane extract of *P. chrysogenum* mycelia which could inhibit the development of human breast cancer cell lines, MDA-468, MDA-MB-231, SK BR-3, BT-474, MCF7, and MCF7-dox, while comparable therapeutic doses revealed no impact on the growth of the non-carcinogenic human mammary epithelial cells MCF10A and viability. Additionally, its therapy inhibited the HGF-induced cell migration and invasion in breast cancer cell lines in a dose-dependent behavior.

Preliminary assay by An et al. (2013) applied for the crude extract of *P. chrysogenum* EN118, separated from the marine brown alga *Sargassum palladium,* showed a weak cytotoxic activity, while 2-(4-hydroxybenzyl)quinazolin-4(3H)-one (98), N-[2-(4-hydroxyphenyl)acetyl]formamide (100) and N-[(2E)-(4-hydroxyphenyl)ethenyl]formamide (96) showed moderate activities against Du145, HeLa and A-549 cell lines with the IC_50_ values of 8, 20, and 20 mg/mL, respectively.

Bladt et al. (2013) stated that chloctanspirone A (150) inhibited human leukemia HL-60 and lung cancer cell line A-549 cell lines with IC_50_ values of 9.2 and 39.7 µM, respectively, while chloctanspirone B (151), showed no activity against the same cell lines.

### Antimicrobial

According to Newaz et al. (2022), several compounds were isolated from the Indonesian mangrove sediment-derived fungus *P. chrysogenum* ZZ1151. The new peniprenylphenol A (200) was found to possess promising antimicrobial activity towards the human pathogens MRSA, *E. coli* and *C. albicans* with MIC values of 6, 13, 13 mg/mL, respectively. In addition, the other known isolated compounds, preparaherquamide (203), uridine (205) and 4-hydroxybenzeneacetic acid methyl ester (207) revealed antimicrobial activity with MIC values in a range from 3 to 25 mg/mL towards the three pathogens. Meanwhile thymine (204) and clavatol (206) demonstrated antibacterial activity against MRSA and *E. coli* only with MIC values of 13–25 mg/mL and 2-hydroxyphenylacetic acid methyl ester (209) showed activity against both MRSA and *C. albicans* with MIC values of 13 and 7 mg/mL, respectively. Also, penicimumide (201) showed antibacterial activity against *E. coli* (13 mg/mL), communol G (102) and 4-hydroxyphenylethanone (210) had activity against MRSA (MIC: 25 mg/mL) and 2,5-dihydroxyphenylacetic acid methyl ester (208) exhibited antifungal activity against *C. albicans* (MIC = 25 mg/mL).

Orfali et al. (2022) investigated compounds (177–181) obtained from Wadi Lajab sediment-derived fungus *P. chrysogenum* for their antimicrobial activity with comparison with five types of pathogenic bacteria *Staphylococcus aureus, Bacillus licheniformis, Escherichia fergusonii, Enterobacter xiangfangensis,* and *Ps. aeruginosa*. All the samples except 6-hydroxymellein (179) revealed selective activities towards Gram-positive bacteria *Staph. aureus* and *B. licheniformis* with MIC values range 0.8 to 21.6 μg/mL. However, 4-chloro-6-hydroxymellein (180) displayed highly potent effect towards Gram-positive bacteria, with MIC 1.00 and 0.8 µg/mL^−1^ for *Staph. aureus* and *B. licheniformis*, respectively.

Qiao et al. (2020) isolated 20β-carboxyl conidiogenone K (29) and 19α-hydroxy conidiogenone C (1) from *P. chrysogenum* TJ403-CA4 extract obtained from the therapeutically valuable arthropod *Cryptotympana atrata*. It was found that they were active against MRSA with MIC values of 4.0 and 2.0 μg/ mL, respectively. Additionally, they revealed good activity towards ESBL-producing *E. coli* and *E. faecalis* with MIC values of 32 μg/mL.

Xu et al. (2020) evaluated the antimicrobial effects of the compounds obtained from the deep-sea algal-derived endophytic fungus *P. chrysogenum* strain XNM-12. It was found that oxalicine C (63) and penicierythritol A (221) revealed moderate antibacterial activity towards the plant pathogen *Ralstonia solanacearum* with MIC values of 8 and 4 μg/mL, respectively.

A study by Chang et al. (2019) on tyrosol (242) isolated from *P. chrysogenum* DXY-1, obtained from deep-sea sediments nearby the East Sea, found that tryosol had an anti-quorum sensing (anti-QS) activity. All studies implied that tyrosol (242) may act as a possible inhibitor for the QS systems to resolve the frightening crisis of bacterial resistance. It may be used as a QS inhibitor against *C. violaceum* and *Ps. aeruginosa.* The docking outcomes showed that it inhibited the QS system of CviR in *C. violaceum* through binding to the DNA-binding domain and blocking pathogenic gene expression**.**

Zhen et al. (2018) treated chrysoxanthones A-C (161–163) obtained from the *P. chrysogenum* HLS111 strain with the histone-deacetylase inhibitor VPA. They were examined against *Staph. epidermidis* (ATCC 12,228, MSSE), *B. subtilis* (ATCC 63,501), *Staph. aureus* (ATCC 29,213, MSSA), *Enterococcus faecalis* (ATCC 29,212, VSE), and *E. coli* (ATCC 25,922). They showed the maximum antibacterial effects against *B. subtilis* with a MIC of 5–10 µg/mL, while they exhibited modest activities towards *Staph. epidermidis* and *Staph. aureus* with MICs of 10–80 µg/mL.

Lopes et al. (2013) reported that the culture filtrates of *P. chrysogenum* IFL1antimicrobial activity showed that the cheese whey culture filtrate inhibited the growth of the *Staph. aureus, Ps. aeruginosa* and *B. cereus.*

An et al. (2013) isolated chrysotriazoles A and B (94–95) from *P. chrysogenum* EN118, an endophytic fungus culture extract isolated from the marine brown alga *Sargassum pallidium*. Its antibacterial activity towards two bacteria, *E. coli* and *Staph. aureus* was assayed, however, none of them showed any inhibitory activity.

### Antioxidant

According to a study by Al-Saleem et al. (2022), Kojic acid (156) showed a potent antioxidant activity with IC_50_ 33.7 ± 0.8 µg/mL compared to the *P. chrysogenum* extract, which was nearly inactive as revealed by the DPPH free-radical-scavenging technique.

Various antioxidant activity techniques were utilized by Jakovljevic et al. (2014), including DPPH free-radical-scavenging activity, Fe2 + -chelating ability, Fe3 + -reducing power and total antioxidant activity. *P. chrysogenum* ethanolic extract which was isolated from wastewater, was found to contain higher total phenolic content and better total antioxidant capacity along with ferrous ion chelating ability.

An et al. (2013) isolated chrysotriazoles A and B (94–95) from *P. chrysogenum* EN118, an endophytic fungus culture extract isolated from the marine brown alga *Sargassum pallidium*. Its radical-scavenging activity was evaluated by the 2,2-diphenyl-1-picrylhydrazyl (DPPH) assay but did not show any activity.

### Miscellaneous

Results of Li et al. (2023) demonstrated that compounds (1, 53 and 197) had antithrombotic activity. Compared to the model group, cerebroside A (197) exhibited substantial antithrombotic activity at the concentration of 25, 50 and 100 μg/mL; ethyl formyltyrosinate (53) had antithrombotic activity at 50, 100 μg/mL; conidiogenone C (1) revealed antithrombotic activity at 50 μg/mL and resulted in zebrafish death at 100 μg/mL.

According to Han et al. (2020), the protein tyrosine phosphatase 1B (PTP1B) enzyme was a confirmed biological target for treating Type II diabetes mellitus for its negative regulatory effect towards insulin signaling cascade. All the isolated compounds obtained and identified from the fungus *P. chrysogenum* SCSIO 07,007, were assessed for their enzyme inhibitory activities against (PTP1B). Also, the new compounds chrysopyrones A and B **(**158 and 159**)** showed noticeable inhibitory activities towards PTP1B with IC_50_ values of 9.32 and 27.8 μg/mL, respectively.

Qi et al. (2020) reported penicichrysogene A and B (16–17), from the substrate culture of *P. chrysogenum* MT-12, an endophytic fungus separated from the medicinal plant of *Huperzia serrata*. The antiplatelet activities were evaluated by applying D. S. Kim’s technique. Penicichrysogene A (16) exhibited antiplatelet aggregation activity with IC_50_ value of 42.7 ± 3.5 μM.

According to Hou et al. (2019) study, the coral-derived *P. chrysogenum *strain (CHNSCLM-0003) yielded chrysogeamides A and B (232–233) which obviously supported angiogenesis in zebrafish at concentration 1.0 μg/mL with nontoxic effect to embryonic zebrafish at concentration 100 μg/mL.

Wang et al. (2018) isolated the compounds (75–77), (195) and (247–263) (246–262) from a deep-sea-derived fungus *P. chrysogenum* SCSIO 41,001, which were tested for their α-glucosidase inhibitory activity by PNPG method. Nine compounds (257, 248, 195, 250, 252, 253, 256, 260, and 262) demonstrated inhibitory action against α-glucosidase with IC_50_ values of 0.35, 0.20, 0.04, 0.16, 0.15, 0.09, 0.14, 0.14, and 0.12 μM, respectively (IC_50_ 0.28 μM for the standard acarbose).

## Conclusions

By revising the existing literature, a massive library of secondary metabolites was isolated and identified, and they also possessed a unique structure. Up to 277 compounds with a variety of structures that belong to various chemical classes were reported from the *Penicillium chrysogenum* endosymbiotic fungus. The chemical structures were classified mainly as terpenoids (majority), alkaloids, polyketides, steroids, flavones (minority), and miscellaneous compounds. *Penicillium chrysogenum* secondary metabolites possess valuable and interesting pharmacological activities, such as antimicrobial, antifungal, cytotoxic, and miscellaneous. This review may be considered a valuable reference for promising pharmaceutical applications or further needed studies on *P. chrysogenum.* Reviewing such an endophyte metabolic pathway on *P. chrysogenum* would help to broaden the future search and discovery of several hundred novel bioactive endosymbiotic compounds with the potential for use as therapeutics. From a global perspective, the endosymbiotic metabolites remain very active and now seem to have the necessary momentum to provide additional antimicrobial, anticancer, and antifungal compounds to the marketplace soon.

## Data Availability

The authors confirm that the data supporting the review are available within the article.
